# Systematic Literature Review of Attempted Suicide and Offspring

**DOI:** 10.3390/ijerph15050937

**Published:** 2018-05-08

**Authors:** Ingeborg Lunde, Marte Myhre Reigstad, Kristin Frisch Moe, Tine K. Grimholt

**Affiliations:** 1 Regional Centre on Violence, Trauma and Suicide Prevention, Region East, 0405 Oslo, Norway; ingeborg.lunde@rvtsost.no; 2 Division of Gynaecology and Obstetrics, Oslo University Hospital, 0424 Oslo, Norway; mfmy@ous-hf.no; 3 Regional Centre on Child and Adolescent Mental Health, Oslo University Hospital, 0424 Oslo, Norway; moe_kristin@yahoo.no; 4 Department of Acute Medicine, Oslo University Hospital, 0424 Oslo, Norway; 5 Department of General Practice, Institute of Health and Society, University of Oslo, 0450 Oslo, Norway

**Keywords:** children, intervention, offspring, parents, suicide attempt

## Abstract

*Background*: Exposure to parental suicide attempt is associated with higher risks of adverse outcomes like lower educational performance, drug abuse and delinquent behavior. When a patient is hospitalized after a suicide attempt, this presents a unique opportunity to identify whether the patient has children, and thereby provide adequate follow-up for both the parent/patient and their children. The objective of this paper was to review the existing literature on follow-up measures for children subjected to parental suicide attempt. *Methods*: In line with the Preferred Reporting Items for Systematic Reviews and Meta-Analyses (PRISMA) statement, we conducted a systematic literature search. *Results*: The search resulted in a total of 1275 article titles, of which all abstracts were screened. Out of these, 72 full text papers were read, and a final four articles were included. Three of the included papers described parts of the same study from an emergency department in The Hague, where a protocol was implemented for monitoring and referring children of parents attempting suicide. The fourth article described the association between maternal attempted suicide and risk of abuse or neglect of their children. *Conclusions*: The lack of research in this particular area is striking. The circumstances surrounding a parent’s suicide attempt call for appropriate familial care.

## 1. Introduction

### 1.1. Experiencing a Parental Suicide Attempt

There are no prevalent figures of how many children are exposed to a parent’s suicide attempt annually. Cerel found that 23% of children with a parent being treated in the emergency department after attempting suicide had been present and witnessed the episode [1]. In a study of adverse childhood experiences, four percent of the 13,494 adults that answered a questionnaire reported a parental suicide attempt [2]. 

For a child it is not only potentially traumatic to experience a parental suicide attempt in itself, but it could also entail living on with the uncertainty and a fear of re-experiencing that the primary caregiver can “choose” to die and leave you. Empirical literature has only described this situation for adult family members and commonly reports constant worrying, being on guard day and night, taking responsibility for activities of daily living and trying to create a nurturing environment for the suicidal relative [3]. Furthermore, they try to prevent a reattempted suicide and perceive this as a significant burden [4,5]. It is likely that children with parents that attempt suicide share these experiences.

### 1.2. Risk Is Associated with Parental Psychiatric Disorders 

A suicide attempt is often associated with psychiatric problems, drug abuse and psychosocial problems [6]. A literature review found that more than 80% of admitted patients had a psychiatric diagnosis [7]. Five percent reported that they were a single parent [8], and children growing up in a single household have been shown to have increased risk for serious suicide ideation [9].

Children from homes with an affectively ill parent are more likely to exhibit general difficulties in functioning, increased guilt, and interpersonal difficulties, as well as problems with attachment. Among these, parental marital difficulties, parenting problems, and severity of parental affective illness are associated with poorer prognosis. Life table estimates indicate that by the age of 20, a child with an affectively ill parent has a 40% chance of experiencing an episode of major depression [10]. Children of depressed mothers are also more likely to report suicidal thoughts [11].

Parental psychiatric history constituted a substantial risk factor for suicide in young people, in particular if the mother was hospitalized for psychiatric illness [12]. The findings are supported by a study that showed an overall suicide attempt rate of 7.8% in the children of depressed parents as compared with 1.4% in the children of non-depressed parents [13]. Further studies have shown that adolescents are almost twice as likely to make a suicide attempt if they have at least one biological parent with mental health problems [14].

In addition to these childhood environmental exposures, it is also conceivable that personality traits like, e.g., hopelessness, neuroticism, and extroversion that are known predictors of suicidal behavior, are transferred from parent to child [15].

### 1.3. Risk Factors and Transmission of Parental Suicidal Behaviour

Children exposed to parental suicidal behavior are four times more likely to report a lifetime suicide attempt compared with unexposed children [16]. Adjusting for confounders including parental depression, maternal suicide attempt is associated with a three-fold increased risk of self-harm with suicidal intent [17], increased risk of multiple attempts [18] and a tendency for early suicide attempts [19].

Adolescents who had experienced the suicide attempt of a family member were also more likely than those with no exposure to report cigarette and marijuana use, alcohol misuse, suicidal ideation and attempts, fighting and inflicting injuries, decreased life-expectancy, emotional distress, and decreased adolescent reports of parent–child and family connectedness [1].

In a Swedish case control registry study, low age at childhood exposure to parental risk factors was associated with increasing risks of suicide and attempted suicide. Adjusted suicide risk was most pronounced in the youngest exposed for parental psychiatric disability pension (3.1), parental attempted suicide (2.9) and suicide (2.9) [20].

In another Swedish population-based study of 14,440 individuals hospitalized due to suicide attempt demonstrated that the strongest independent familial risk factors for youth suicide attempt were siblings’ (OR 3.4), maternal (OR 2.7) and paternal (OR 1.9) suicide attempt [21]. Other identified risk factors associated with parental suicidal behavior are diminished educational performance [22], as well as substance abuse, e.g., illicit drugs, tranquilizers and opioids [23].

Neglecting parenting and sexual-abuse are increased among children of patients with attempted suicide [24] and increased risk for suicidal behavior [25,26,27]. In turn, both neglecting parenting and being sexually abused in addition to experiencing parental suicidal behavior increases risk of suicide attempt at a young age [28]. 

Summarizing the findings above, childhood exposures to parental suicidal behavior is linked to a variety of negative behavioral and health-related outcomes. This underpins attempts at early identification and interventions in families at risk, to prevent these factors’ impact.

People who have attempted suicide themselves struggle with severe problems and need help [29]. If it is hard to cope with one’s own psychosocial problems, needless to say, the ability to seek help for one’s own children is probably impaired.

Not everyone who attempts suicide has been treated in the health care services previously [8,30], and hospitalization is therefore an opportunity to identify whether they have children at home in need of immediate measures, long-term follow-up or both. Also, it is crucial to assess and re-evaluate any on-going treatment; whether there is a need to extend levels of care and cooperation between services, for both the patient and their children. Furthermore, do we know what factors should elicit concern among health care providers, which questions should be asked and what kinds of interventions are ideal for these families?

### 1.4. Review Questions

(a) Do guidelines (advice for health care personnel in order to assure quality of the services) and routines (fixed program or regularly actions) exist to screen for and follow-up children of parents attempting suicide?
(b) Are there interventional studies aimed at treating or following up children after a parent has attempted suicide?

## 2. Methods

### 2.1. Protocol and Registration

The protocol for this systematic review was registered in PROSPERO. ID CRD42017051109. (http://www.crd.york.ac.uk/PROSPERO/display_record.asp?ID=CRD42017051109).

### 2.2. Eligibility Criteria 

The Preferred Reporting Items for Systematic Reviews and Meta-Analyses (PRISMA) guidelines were followed when we performed the literature search and prepared the manuscript [31]. 

We searched for randomized controlled clinical trials to assess the beneficial effects of the treatments, and included any observational studies (cohort and case–control studies). To assess whether there are guidelines for identification and clinical measures, relevant study designs included qualitative studies.

The Patient Population or Problem, Intervention, Comparison (group or treatment), Outcomes and Setting (PICOS) criteria used are listed below [32].

P: children of patients with suicide attempt, parasuicide, deliberate self-harm or self-harm.

I: any kind of intervention for the child of the patient or both patient and child (e.g., family oriented interventions).

C: children that did not receive the intervention, or other forms of treatment. 

O: any type of health-related and psychosocial outcome in childhood, adolescence or young adulthood.

S: all study settings used to identify offspring of patients admitted to any treatment institutions with a suicide attempt, deliberate self-harm or self-harm.

### 2.3. Data Sources and Search Strategy

A librarian helped one of the authors (Tine K. Grimholt) to perform the database searches.

Articles in English, Danish, Swedish and Norwegian language were included, and there were no limitations regarding year of publication. We searched in PubMed (included papers not complete registered with medical sub heading terms), PsycINFO and Cochrane Database of Systematic Reviews. 

In addition we searched through ORIA—the Norwegian library portal—for all relevant titles among electronical journals, books and published papers as well as international master and doctoral theses.

Bibliographies from the following guidelines were searched for additional publications: The Norwegian Health Library for summarized research, National network for procedures (Norway), Sundhetsstyrelsen (Denmark), Danish Centre for clinical guidelines, Socialstyrelsen (Swedish national guidelines), and Swemed-plus (Swedish research database), as well as UpToDate, Best Practice, NICE Guidance (UK), and National Guideline Clearinghouse. 

Further, we searched for registered ongoing trials in Clinicaltrials.gov and reviews in PROSPERO. 

The following search string was used to search for papers published in English:

((“Suicide, Attempted” [Mesh] OR “Self-Injurious Behavior” [Majr:NoExp] OR “Suicide” [Mesh:NoExp] OR “Suicidal Ideation” [Majr]) AND (“Child of Impaired Parents” [Mesh] OR Parents [majr] OR Maternal Behavior [majr] OR Paternal Behavior [majr] OR Parent-Child Relations [majr] OR Paternal Deprivation [majr] OR Maternal Deprivation [majr] OR Parental Death [majr] OR Parenting [majr] OR “Family” [Majr:NoExp] OR “Adult Children” [Majr] OR “Family Relations” [Majr:NoExp] OR “Family Conflict” [Majr] OR “Professional-Family Relations” [Mesh] OR “Caregivers” [Majr]) AND (Danish [lang] OR English [lang] OR Norwegian [lang] OR Swedish [lang])) OR ((suicide [ti] OR suicides [ti] OR suicidal [ti] OR self-Injurious [ti] OR self-harm [ti] OR self-poisoning [ti] OR parasuicide [ti] OR parasuicides [ti]) AND (offspring [ti] OR family [ti] OR families [ti] OR relatives [ti] OR caregiver [ti] OR caregivers [ti] OR carer [ti] OR carers [ti] OR mother [ti] OR mothers [ti] OR father [ti] OR fathers [ti] OR maternal [ti] OR paternal [ti] OR parent [ti] OR parents [ti] OR parenting [ti]) AND (Danish [lang] OR English [lang] OR Norwegian [lang] OR Swedish [lang]) NOT medline [sb]).

During the work with this paper we also unsystematically contacted and discussed relevant literature with researchers who have broad experience from the field of suicidology. No one had knowledge of any additional literature or research relevant for the review questions.

### 2.4. Study Selection

The search through Pub Med generated 1174 original papers published in peer-reviewed scientific journals per 20 October 2016. In addition, 101 titles were obtained from Psych Info and Swemed-plus, resulting in a total of 1275 scientific articles. All these abstracts were screened, out of which 72 full-text original papers were selected and assessed for eligibility. 

**Figure 1:**
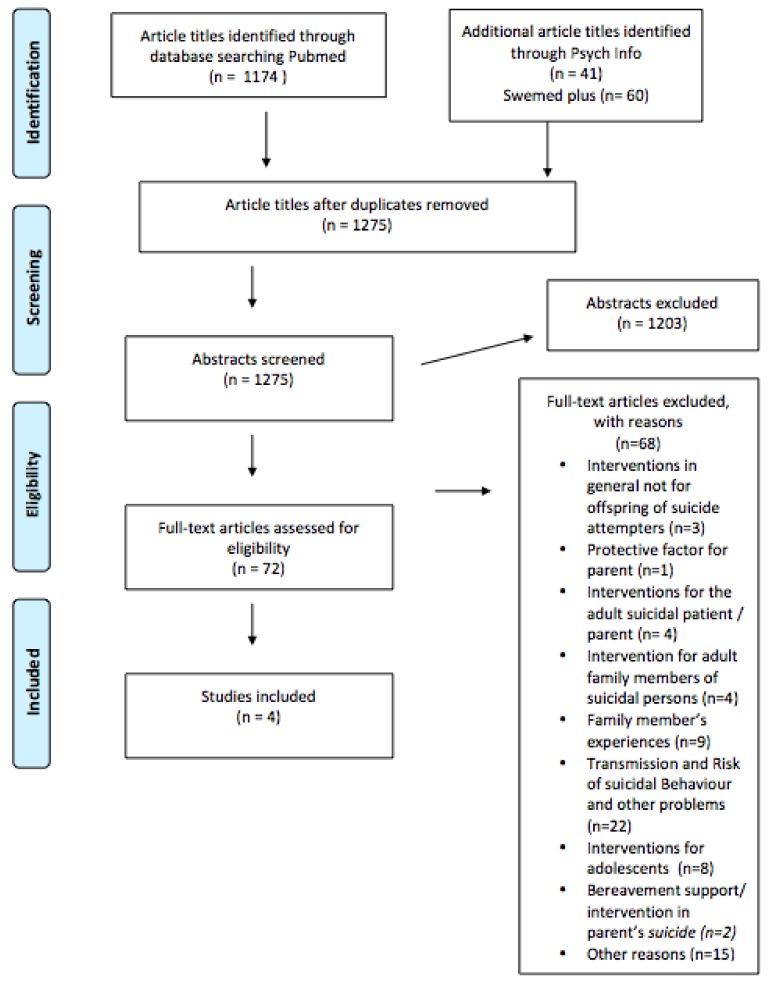
Preferred Reporting Items for Systematic Reviews and Meta-Analyses (PRISMA) Flow Diagram.

Of these, 68 were excluded as they pertained to bereavement support/intervention in parent’s suicide (*n* = 2) [33,34], interventions for adolescents (*n* = 8) [35,36,37,38,39,40,41,42], transmission and risk of suicidal behavior and other problems (*n* = 22) [1,11,12,16,17,19,25,28,43,44,45,46,47,48,49,50,51,52,53,54,55,56], other reasons (*n* = 15) [57,58,59,60,61,62,63,64,65,66,67,68,69,70,71], family member’s experiences (*n* = 9) [3,4,5,72,73,74,75,76,77], interventions for adult family members of suicidal persons (*n* = 4) [78,79,80,81], interventions for the adult suicidal patient/parent (*n* = 4) [82,83,84,85], whether having a child was a protective factor for the suicidal parent (*n* = 1) [86] and finally interventions and preventive measures in general not for children of a parent who has attempted suicide (*n* = 3) [87,88,89] (Figure 1).

A final total of four eligible papers were included in the review.

The search for clinical guidelines, registered trials, systematic reviews and other relevant literature yielded two findings. In one of the guidelines, caring for a minor child was described in relation to whether it was a protective factor for the parent’s further suicidal behavior. 

https://www.uptodate.com/contents/suicidal-ideation-and-behavior-in-adults?search=suicide%20attempt&source=search_result&selectedTitle=1~150&usage_type=default&display_rank=1.

In the Nice guidelines of long term treatment and management of self-harm, it is recommended that risk of domestic or other violence or exploitation should be considered along with local safeguarding procedures for vulnerable adults and children in their care. 

https://www.nice.org.uk/guidance/CG133/chapter/1-Guidance#longer-term-treatment-and-management-of-self-harm.

Tine K. Grimholt and Ingeborg Lunde read the abstracts, full texts and the four papers that were selected and worked together to construct the table and verify the data within them.

## 3. Results

The first three papers were studies conducted as part of the “Hague Protocol” in The Netherlands, by the same research group (Table 1). The main objectives of these studies were to detect child abuse among parents admitted to emergency departments based for domestic violence, substance abuse or suicide attempt/self-harm. Families were referred to a Reporting Center for Child Abuse and Neglect to enable rapid assessment of family problems and those in need subsequently offered voluntary community-based support.

The first paper aimed to investigate whether parents avoided medical care if they were included in the above protocol. The results showed that the parents did not avoid medical care if referred. There was no decline in the number of patients included [90].

The second paper aimed to assess whether The Hague protocol for screening adults presenting for care in the Emergency Department could identify children at high risk for maltreatment. It employed a case–control (before–after) design in nine emergency departments distributed on one intervention region and two control regions. The protocol had a high positive predictive value of 91% and increased the detection rate of child abuse. In the sample, 89% of the referrals substantiated child abuse and of these 80% were newly identified [91]. 

The third paper investigated what had happened to the families three months after referral to the Reporting Centre for Child Abuse and Neglect. Of the 99 cases where information was available, existing support was continued or intensified in 31, a Child Protection Services (CPS) report had to be made in 24, new support was organized for 27 cases and in 17 cases support was not necessary, because the domestic problems were already resolved. In the sample there were 22 parental suicide attempts. Of these, nine children had experienced educational neglect, four psychological violence, two emotional neglect. One had witnessed domestic violence. Four had experienced combinations of these types of maltreatment and referral was not substantiated in two cases [92].

The fourth paper included in this review is from the UK and conducted by Hawton and colleagues in 1985. They studied the association between attempted suicide in 114 mothers admitted to a general hospital and child abuse in children aged five years and below. A case-control design was used, and documented increased risk of child abuse among 29.8% of those who attempted suicide, compared to mothers at risk for depression and to the general population [24].

## 4. Discussion

### 4.1. Summary of Evidence

In spite of the large body of evidence dating back to the mid 1960s that clearly demonstrates parental suicidal behavior to be a significant risk factor for several adverse outcomes in children, interventional studies are lacking.

Although no limitations were placed on study quality, we only found four studies that addressed children of parents hospitalized with a suicide attempt. On account of the scarcity of data, no recommendations for clinical care can be made. 

This important research gap in the field of suicidology should be highlighted, and particularly the lack of interventional studies in contrast to the vast majority focusing merely on looking for risk factors. 

The recently updated Cochrane reviews of interventions for adults and children and adolescents with deliberate self-harm and suicide attempts [93,94], demonstrate that the amount of interventional research is scant. There were 55 interventional studies (randomized controlled trials) for adults, and 11 for children and adolescents. However, not one of these studies on adults included interventions for children who were next of kin.

In summary, this should prompt researchers, health care providers and policy makers to focus on singling out and offering intervention to a large and highly vulnerable group of children.

There is a possibility that the methods we used in this review did not enable us to retrieve and identify all research.

There were astonishingly few studies aimed at intervening to help children with parents that had attempted suicide. This may reflect the difficulties in designing interventional studies to prevent transmission of suicidal behavior from parent to child. However, there are several examples of longitudinal prospective studies on long-term outcomes. This is demonstrated by a prospective study by Conner and colleagues that examined the parent-to-child “transmission” of risk for suicide attempt. The researchers included diagnostic interviews with parents and children, and examined the transition from childhood (nine years) to adolescence (18 years) [95].

Furthermore, Reider and Sims described beneficial cross-over effects from four studies not originally designed to measure suicidal behavior as outcome, but were able to provide additional data. The common factors in these studies were that they all promoted healthy parent–child interactions [87]. It seems promising that promotion of adaptive parent–child relationships and behaviors can protect against negative developmental trajectories [88].

As demonstrated by Felitti, there was a graded relationship between the amounts of exposure to abuse or household dysfunction during childhood and multiple risk factors for several of the leading causes of mortality in adults [2]. The prevalence of risk factors such as child neglect and substance abuse disorders are elevated among patients admitted to hospital with suicide attempts [7,24]. Further, as demonstrated by Diderich et al., the vast majority of child abuse was detected among the patients during the first time when they were hospitalised [91].

Based on these studies, it may be reasonable to design specific interventions targeted at improving attachment and familial management, as well as reducing the environmental exposure of risk factors. Along these lines, interventions for children with mentally ill parents have shown some promising results [96].

Brent suggested that children of parents with depression or substance abuse should be psychiatrically screened and that family interventions to decrease discord would be helpful in decreasing the risk of adolescent suicide [51]. Similarly, we now suggest that children of parents with suicide attempt should be screened and receive interventions.

### 4.2. Limitations

The samples in the included studies in our review were patients admitted to a general hospital after a suicide attempt; however, the studies using The Hague protocol also included patients who were hospitalized for domestic violence and abuse. Based on these studies, it is not possible to conclude whether screening and referral from the emergency ward is sufficient or reduces adverse outcomes among the children at risk. 

The Hague protocol was the only initiative described in research literature, and the design should be conducted in further large-scale studies and preferably as a randomized controlled clinical trial, where outcomes like the post-discharge risk factors described in this paper are included.

Although the search strategy in the current review was comprehensive, we may have missed data included in local guidelines developed in hospitals. In Norway, for example, the government has recently developed initiatives for awareness and focus on children as next of a kin. A law that incorporates this group of children’s rights to information and proper care was amended in 2010. 

We might also have missed literature published in other languages than Scandinavian and English and grey literature that could have been discovered by using Google Scholar and research published after the literature search.

### 4.3. Consequences Practical Implications

A substantial proportion of deliberate self-harm patients discharged directly from acute and emergency departments do not receive a psychiatric assessment [97] and it is therefore important to have guidelines for screening. In a stressful clinical schedule, it is not necessarily enough to trust that the clinicians and especially the younger and less experienced clinicians remember and prioritize this. 

Suicidal behaviors occur as a result of complex interactions between social factors, psychiatric illness and environmental influences. It is important to focus the research into early interventions in families at risk in order to prevent suicidal behavior and other adverse outcomes.

Furthermore, insecure parental attachment merits further study investigation as a potential target in order to reduce the risk of children’s psychopathology and suicidal behavior [55].

## 5. Conclusions

We found almost no studies about interventions that include the children of patients hospitalized with a suicide attempt. Clinical implications of our findings include a need for awareness and cooperation between healthcare professionals to facilitate detection and potential need for intervention in these children. Efforts should be made by researchers to identify measures to prevent the familial transmission of suicidal behavior. This would in turn reduce adverse outcomes, morbidity and mortality of suicidal behavior in a well-documented high-risk group of youths. 

## Figures and Tables

**Table 1:** Overview of the studies included in the review.

Country Year Reference	Design	Setting/Participants	*n*	Intervention	Outcome	Results
Netherland 2015 [90]	Case–control Study (before-after) & qualitative design Parents (*n* = 14) interviewed by telephone	Emergency department Parents admitted for -domestic violence -substance abuse or -suicide attempt or self-harm.	14	Referral made to the Reporting Center for Child Abuse and Neglect (RCCAN).	Does parents avoid medical care?	Parents don’t avoid medical care if referred No decline in the number of patients, included in the Protocol
Netherland 2015 [91]	Case Study	Emergency department referrals based on parental characteristics in which child abuse was confirmed after investigation by the RCCAN were analysed	100 (99)		Consequences for the families three months after referral: Type of child abuse, reason for reporting, duration of problems prior to the ED referral, previous involvement of support services or other agencies, re-occurrence of the problems and outcome of the RCCAN monitoring l.	Existing support continued or intensified *n* = 31 Child Protection Services report made *n* = 24 New support organized *n* = 27 Support not necessary *n* = 17 Not followed up *n* = 31.
Netherland 2013 [92]	Case-control Study (before-after)	Nine Emergency departments in 3 regions (one intervention region and 2 control regions). From January 2006 to November 2007		Screening and Reporting to Centre for Child Abuse and Neglect (RCCAN) to assess family problems and offer voluntary community based support to parents.	Referrals to the Centre for Child Abuse and Neglect	Before implementation of the protocol (1 per 100,000) After implementation of the protocol (64 per 100,000). In the control region (1 per 100,000) and (3 per 100,000) (OR = 28.0 (95 CI 4.6–170.7)).
United Kingdom 1985 [24]	Case-control Study	Mothers with children aged five years and under admitted to General hospital for attempted suicide	114	No	Child abuse	Risk of child abuse was identified in 29.8% of those who attempted suicide.
